# Anesthetic management of pulmonary artery replacement for idiopathic dilatation of the pulmonary artery with a Kommerell’s diverticulum: a case report

**DOI:** 10.1186/s40981-020-00404-w

**Published:** 2021-01-04

**Authors:** Akira Nishioka, Kyosuke Takahashi, Yasuhiro Maehara

**Affiliations:** 1 Department of Anesthesiology, Kohnodai Hospital, National Center for Global Health and Medicine, 1-7-1 Kohnodai, Ichikawa, Chiba, Japan; 2 Department of Anesthesiology and Critical Care, Jichi Medical University Saitama Medical Center, 1-847, Amanumacho, Omiya, Saitama, Japan; 3 Department of Anesthesiology, National Center for Global Health and Medicine, 1-21-1, Toyama, Shinjuku, Tokyo, Japan

**Keywords:** Idiopathic dilatation, Dilated pulmonary artery, Anesthetic management, Preoperative evaluation, Pulmonary artery replacement

## Abstract

**Background:**

Idiopathic dilatation of the pulmonary artery (IDPA) is a rare condition in which the pulmonary artery dilates without an obvious cause. Pulmonary artery replacement is indicated in severe cases to prevent serious complications.

**Case presentation:**

A 59-year-old man was diagnosed with an IDPA of 64 mm and Kommerell’s diverticulum (aortic aneurysm located at the aberrant left subclavian artery). A computed tomography scan revealed slight compression of the aneurysm to the trachea, although not interfering with airway management. The surgical approach was a median sternotomy, and cardiopulmonary bypass was established through aortic and bicaval cannulations. The perioperative course was uneventful.

**Conclusions:**

To prevent injury to the dilated pulmonary artery, a strategy for cardiopulmonary bypass and a surgical approach should be discussed beforehand. As dilatation of the pulmonary artery is often complicated by anatomic abnormalities, preoperative evaluation should be aimed at appropriate assessments using imaging modalities.

## Background

Idiopathic dilatation of the pulmonary artery (IDPA) is a rare condition characterized by enlargement of the pulmonary artery in the absence of an obvious trigger with an incidence of 1 per 14,000 autopsies [1]. The diagnostic criteria for IDPA are (1) diastolic change of the pulmonary artery mainstem; (2) no abnormal shunt inside or outside the heart; (3) no lesions of the arteries that could cause histological changes, such as syphilis and arteriosclerosis; (4) no chronic heart or lung disease; and (5) normal right ventricular pressure. Generally, a patient who meets all the above-mentioned criteria is diagnosed with IDPA. Dilatation of the pulmonary artery may be secondary to an underlying disorder, such as congenital heart disease (including patent ductus arteriosus), chronic heart failure, chronic respiratory disease, vasculitis, Marfan syndrome, and trauma. Given the potential complications associated with IDPA, such as rupture of the pulmonary artery [2], pulmonary valve insufficiency [3], and myocardial ischemia due to displacement of the coronary arteries [4], surgery is a valid option for the treatment of this condition. Although there are several reports on surgery for dilated pulmonary arteries, few have explored the feasibility of anesthetic management for IDPA [5]. We present a case of IDPA complicated by an extracardiac abnormality (Kommerell’s diverticulum), and discuss the management of IDPA.

## Case presentation

**Fig. 1:**
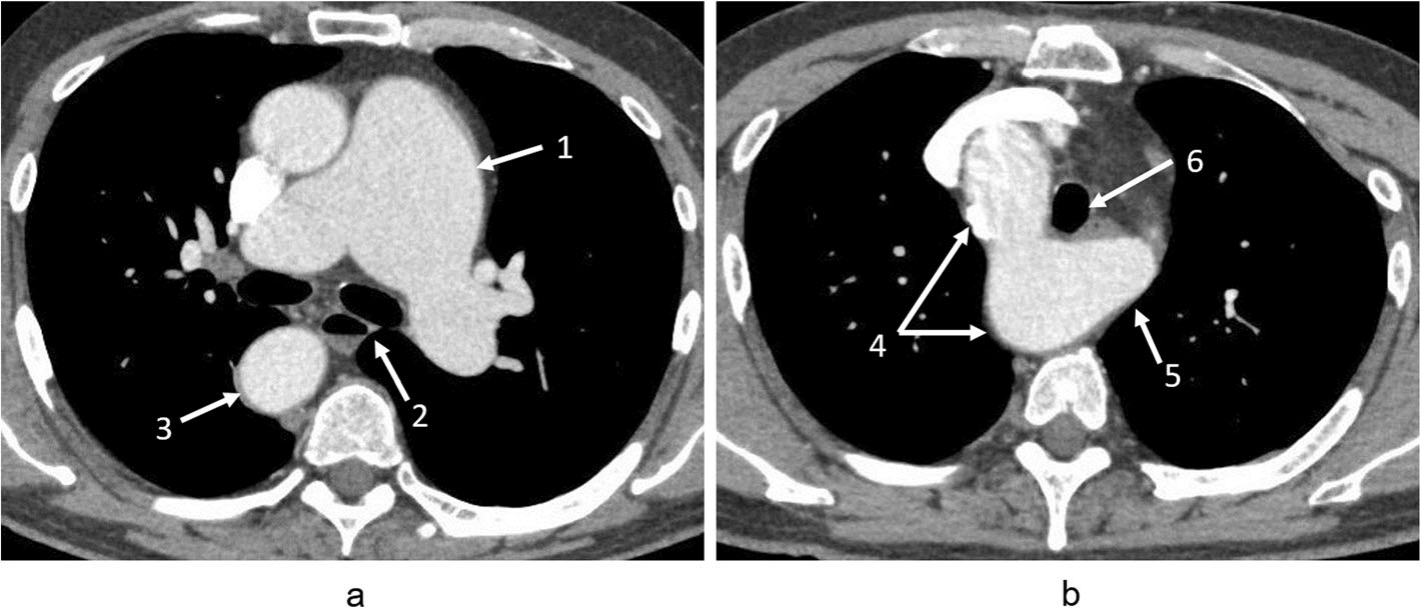
**a** Contrast computed tomography showing a dilated pulmonary trunk. The dilated part of the pulmonary trunk (arrow 1) is not in contact with the sternum, nor does it displace the mediastinal tissues (arrow 2). The descending aorta is depicted in the right posterior mediastinum (arrow 3). **b** Arrow 4 indicates the right-sided aortic arch. The Kommerell’s diverticulum (3 cm) is depicted at the origin of the left subclavian artery (arrow 5). The trachea is slightly displaced by the diverticulum (arrow 6)

A 59-year-old man (height, 167 cm; weight, 71 kg) underwent a medical check-up that revealed an abnormal radiopacity on a chest radiograph. Consequently, he underwent computed tomography (CT), which led to the diagnosis of IDPA. He visited our institution for surgical treatment. CT showed a dilated pulmonary artery measuring 64 mm (Fig. 1a). It also revealed a right aortic arch, featuring an aberrant left subclavian artery with an aneurysm measuring 3 cm (Kommerell’s diverticulum) (Fig. 1b). Transthoracic echocardiography showed normal wall motion and no significant valvular disease. Cardiac catheterization demonstrated normal pulmonary artery pressure and no stenosis in the coronary arteries. Pulmonary artery replacement was planned to prevent rupture of the dilated pulmonary artery aneurysm.

After placement of the radial artery catheter, anesthesia was induced with fentanyl (0.5 mg), propofol (150 mg), and rocuronium (100 mg). The trachea was intubated with an 8.0-mm tracheal tube using a video laryngoscope (McGRATH^TM^, Medtronic, Minneapolis, MN, USA) without difficulty. The mechanical ventilation settings were fraction of inspired oxygen of 60%, frequency of 12 breaths per minute, tidal volume of 6 mL/kg, and positive end-expiratory pressure (PEEP) of 3 cm H_2_O. The target range for end-tidal CO_2_ was set at 30–35 mmHg. A transesophageal echocardiography (TEE) probe was inserted carefully while confirming that there was no resistance. Anesthesia was maintained with sevoflurane (1%) and remifentanil (0.3 μg/kg/min). A pulmonary artery catheter (PAC) was inserted into the right internal jugular vein and carefully advanced under TEE monitoring in the mid-esophageal bicaval view and right ventricular inflow-outflow view. Finally, in the ascending aorta short-axis view, it was confirmed that the catheter tip was in the main pulmonary artery and that there was no malposition or flexion. After PAC insertion, the mean pulmonary arterial pressure was 24 mmHg.

The approach of the surgery was a median sternotomy. Cardiopulmonary bypass (CPB) was instituted through aortic and bicaval cannulation. Main pulmonary artery replacement was performed on a beating heart. The dilated part of the main pulmonary artery was resected. The right and left pulmonary arteries were anastomosed to the edges of a 24-mm graft, and the remaining main pulmonary artery was anastomosed to the side of the graft. The patient was easily weaned from CPB with administration of dobutamine (2 μg/kg/min) and milrinone (1 μg/kg/min). The intraoperative mean pulmonary arterial pressure was consistently maintained at around 20 mmHg. The patient was transferred to the intensive care unit (ICU) under mechanical ventilation. The patient was extubated on the day of ICU admission, with a subsequent uneventful course. Mean pulmonary arterial pressure was consistently maintained at 19–24 mmHg during the ICU stay. The patient was discharged on postoperative day 16.

## Discussion

The diagnostic criteria for dilatation of the pulmonary artery due to an unknown etiology established by Greene et al. [6] and Deshmukh et al. [7] have been used for the diagnosis of IDPA. In the present case, the patient had pulmonary artery dilatation of 64 mm in the pulmonary artery stem, but no medical history indicative of a potential cause. Additionally, transthoracic echocardiography did not suggest evident valvular disease or intracardiac shunting in the right ventricle system. Consequently, the patient was diagnosed with IDPA. Typically, IDPA is a condition that does not cause tissue degeneration of the pulmonary arteries. Correspondingly, it does not increase the pressure of the pulmonary arteries and right ventricle. An occurrence of idiopathic pulmonary artery dilatation is very rare, and few reports on the outcomes of the treatment strategies used for this condition exist. Therefore, there is no clear consensus on indications for surgery to treat IDPA.

On the contrary, there are some reports on dilatation of the pulmonary artery in response to particular precipitating factors [3]: complicating pulmonary regurgitation, rapid growth of the dilated artery, which can lead to dissection and rupture of the pulmonary artery [2], pulmonary incompetence [3], and myocardial ischemia [4]. Thus, dilatation of the pulmonary artery beyond a certain extent may be a factor influencing prognosis. Similar complications are thought to occur in patients with IDPA once the pulmonary artery exceeds the capacity of the mediastinum. Thus, surgery should be performed when dilatation reaches 6 cm or more [8].

In the present case, the procedure for anesthesia management in pulmonary artery replacement for IDPA did not differ substantially from other typical anesthesia management strategies in cardiovascular surgery that use CPB. However, there are two presumed disease-specific critical conditions in anesthetic management: rupture of the dilated pulmonary artery and airway compression.

Surgical injury or rupture of the pulmonary artery is the greatest concern. Particularly, rupture before ensuring CPB can rapidly result in a fatal hemorrhage. Since sternotomy is thought to confer the highest risk of damage to the pulmonary artery, understanding the degree of dilatation of the pulmonary artery and the location and position of the adjacent structures on the basis of preoperative imaging is necessary. For patients whose dilated pulmonary artery is in close proximity to the sternum, CPB via femoral cannulation should be considered to avoid damage caused by sternotomy. Furthermore, intraoperative transient elevation of pulmonary arterial pressure may lead to dissection and rupture of the pulmonary artery [2]. However, for patients with IDPA only, previous reports have indicated that the pulmonary arterial pressure is maintained at a normal or slightly elevated level [9]. Additionally, insertion of the PAC may cause dissection or rupture of the pulmonary artery. Kearney et al. analyzed 32,000 complications of PAC insertion over a period of 17 years; the incidence of pulmonary artery injury associated with catheterization was 3 per 10,000 cases, with a resulting mortality of 70% [10].

Considering these facts, it may not be worthwhile to monitor the pulmonary arterial pressure in the perioperative period for a patient with IDPA. However, in the present case, the surgeon requested PAC placement prior to surgery to monitor perioperative pulmonary arterial pressure. Kearney et al. reported the following risk factors for PAC-associated rupture of the pulmonary artery: (1) age of 60 years or more, (2) pulmonary hypertension, (3) inappropriate balloon dilatation, (4) inappropriate catheter position, (5) use of CPB, and (6) poor anticoagulation [10]. In the present case, PAC placement was performed since it was considered to be relatively low-risk, provided the catheter was inserted appropriately. Moreover, we focused on certain important aspects in order to prevent rupture of the pulmonary artery. During mechanical ventilation, low airway pressure and mild hypocapnia were maintained to avoid raising pulmonary vascular resistance. In addition, we took care to avoid excessive transfusion, and initiated continuous infusion of dobutamine and milrinone prior to weaning the patient from extracorporeal circulation. This strategy may have allowed us to maintain the patient’s pulmonary arterial pressure within the normal range throughout the perioperative period.

Another complication is compression of the airways by the dilated pulmonary artery. Additionally, 50% of patients with pulmonary artery dilatation suffer from some form of congenital heart disease [11]. Our patient had a Kommerell’s diverticulum, which slightly displaced the trachea. Such displacement by an aneurysm or arterial ring may occur with Kommerell’s diverticulum. Regarding induction of anesthesia, administration of a muscle relaxant and intermittent positive-pressure ventilation may change the diameters of the trachea and the bronchus that are damaged by external pressure due to aneurysms [12]. Since not only Kommerell’s diverticulum but also dilated pulmonary arteries may affect airways, it is important to identify potential risk factors that could hinder airway management.

In conclusion, we present a case of IDPA with a Kommerell’s diverticulum treated by pulmonary artery replacement. Regarding the anesthetic management for this procedure, it is particularly important to prevent rupture of the dilated pulmonary artery and anticipate potential difficulties in airway management. A strategy for the surgical procedures and CPB management should be carefully designed, and special attention should be paid to anatomic abnormalities.

## Data Availability

The datasets used and/or analyzed during the current study are available from the corresponding author on reasonable request.

## Abbreviations

CPB: Cardiopulmonary bypass
CT: Computed tomography
ICU: Intensive care unit
IDPA: Idiopathic dilatation of the pulmonary artery
PAC: Pulmonary artery catheter
PEEP: Positive end-expiratory pressure
TEE: Transesophageal echocardiography
